# Systemic Lupus Erythematosus With Hemophagocytic Lymphohistiocytosis: Is COVID-19 the Inciting Factor?

**DOI:** 10.7759/cureus.19657

**Published:** 2021-11-17

**Authors:** Ruhma Ali, Rime Mehannek, Aditya Patel, Amy Paige, Sahithi Reddy, Michael Guma, Gunwant Guron

**Affiliations:** 1 Internal Medicine, Saint Michael’s Medical Center, Newark, USA; 2 Hematology and Oncology, Saint Michael’s Medical Center, Newark, USA; 3 Rheumatology, Saint Michael’s Medical Center, Newark, USA

**Keywords:** autoimmune disease, cytokine storm, covid-19, hemophagocytic lymphohistiocytosis, systemic lupus erythematosus

## Abstract

Systemic lupus erythematosus (SLE) is a chronic autoimmune disease that is characterized by an aberrant immune response leading to immune-mediated damage to tissues. Hemophagocytic lymphohistiocytosis (HLH), a life-threatening condition, consists of a constellation of symptoms caused by excessive immune activation and cytokine storm. HLH is categorized into the primary and secondary form. The secondary form is often referred to as the macrophage activation syndrome. HLH in the background of SLE is a rare and potentially fatal entity. It is often seen in the context of disease flare and is rarely associated with the initial diagnosis of SLE. Severe infection with severe acute respiratory syndrome coronavirus 2 (SARS-CoV-2) causes a cytokine storm characterized by marked elevation of inflammatory markers including ferritin. Here, we describe the case of a young female with an inaugural diagnosis of SLE and features of HLH after a recent SARS-CoV-2 infection.

## Introduction

Hemophagocytic lymphohistiocytosis (HLH) is an underrecognized, life-threatening condition that requires prompt treatment and diagnosis. HLH is categorized into two separate forms. The primary or familial form is autosomal recessive [1], whereas the acquired or secondary form can be due to infectious causes, malignancies, or rheumatological disorders. HLH is characterized by systemic hyperinflammation due to the uncontrolled proliferation of lymphocytes, macrophages, and CD8 T-cells, leading to massive cytokine release. This sequence of events leads to tissue damage and progressive multiorgan failure [2]. The hallmark features of HLH include persistent fevers, pancytopenia, hepatosplenomegaly, hyperferritinemia, and hypertriglyceridemia. HLH in the background of systemic lupus erythematosus (SLE) is uncommon and requires clinical acuity and awareness to avoid complications from the sequelae of the disease. Viral infections are known to trigger secondary HLH. The clinical and laboratory features of secondary HLH and coronavirus disease 2019 (COVID-19) are synonymous, with COVID-19 likely serving as a nidus for unmasking autoimmune diseases.

## Case presentation

A 25-year-old female with a medical history of asthma and depression presented to the Emergency Room with chief complaints of generalized weakness, fatigue, nausea, vomiting, and bloody diarrhea that progressively worsened in the past two months. She had presented to the Emergency Department (ED) two months back for a cough and shortness of breath. At that time, she denied any headache, neck stiffness or pain, sore throat, or chest pain. Her physical examination was normal. Reverse transcription-polymerase chain reaction for severe acute respiratory distress syndrome coronavirus 2 (SARS-CoV-2) was positive. D-dimer was also markedly elevated. Chest X-ray showed no cardiopulmonary abnormality. She did not require hospitalization and was told to home quarantine for two weeks. She did not receive remdesivir, dexamethasone, or anticoagulation for COVID-19. However, she received oseltamivir for five days as she tested positive for the influenza B virus. At the time of the current admission, she complained of a rash on her face and scalp as well as swelling of her tongue and lips. The patient stated that she has been restricted to the bed due to severe muscle weakness and had become irritable and anxious. Her family members noticed a change in her affect and concentration. She acknowledged taking pain medications without any significant relief and denied smoking or alcohol use. A review of the others systems was unremarkable. On admission, she was afebrile, with a blood pressure of 130/80 mmHg, respiratory rate of 28 breaths/minute, heart rate of 101 beats/minute, oxygen saturation of 95% on room air, and body mass index of 28.52 kg/m^2^. On physical examination, the patient appeared weak and tired. She had pale conjunctiva with jaundice. Tenderness was noted on palpation of the sternum and left chest. Abdominal examination was significant for mild epigastric tenderness. Additionally, right inguinal lymphadenopathy was noted. Muscle strength was 3/5 in bilateral lower extremities and 4/5 in bilateral upper extremities. Cranial nerves were grossly intact; however, knee reflexes were not appreciated. A few dry, brownish lesions of 3-5 mm diameter were scattered around the nose. Ulcers were noted on the hard palate, measuring approximately 2 cm in diameter, and on the scalp. Perioral ulceration was also seen.

Initial laboratory investigations revealed elevated triglycerides, lactate dehydrogenase (LDH), fibrinogen, ferritin, and D-dimer with normal C-reactive protein (CRP). Detailed laboratory parameters comparing laboratory findings from the previous ED visit and over the course of hospital stay are listed in Table 1. She had an acute kidney injury with increased creatinine and rhabdomyolysis with total creatine kinase of 3,223 U/L (reference range: 38-176 U/L). Her chest X-ray showed a possible consolidation with pleural effusion. An echocardiogram showed large pericardial effusion. Diagnostic pericardiocentesis was done which was consistent with transudative pericardial fluid from ongoing underlying inflammatory conditions.

**Table 1:** Laboratory parameters. ED: Emergency Department; CRP: C-reactive protein; LDH: lactate dehydrogenase; AST: aspartate transaminase; ALT: alanine transaminase

Laboratory parameters	Previous ED visit (2 months prior)	Admission day 1	In-hospital day 7	Extubation (day 53)	1-week post-extubation (day 60)	Reference range
Hemoglobin (g/dL)	10.4	10.2	8.1	8.8	8.2	12–15.5
Neutrophils (×10^3^/µL)	1.9	1.3	11.1	4.6	3.9	1.7–7
Lymphocytes (×10^3^/µL)	0.8	2.7	0.4	1.3	1.4	0.9–2.9
Platelet count (×10^3^/µL)	109	35	60	92	126	150–450
Creatinine (mg/dL)	0.7	2.4	5	<0.2	0.2	0.6–1.2
CRP (mg/dL)	0.6	<0.3		0.9		0.0–0.8
Ferritin (ng/mL)	143.6	17,500	960.2			11–307
Bilirubin (mg/dL)	0.3	5.7	3.9	0.5	0.3	0.2–1.2
LDH (U/L)	307	2252	1145	405	333	122–222
Fibrinogen (mg/dL)		82	259	169	186	200–393
Triglyceride (mg/dL)	142	405	431			0–150
D-dimer (ng/mL)	1891	7086	7280			0–500
AST (U/L)	35	3831	456	23	14	10–36
ALT (U/L)	18	535	52	35	24	6–29

The patient was admitted to the Intensive Care Unit for close monitoring and further investigation. She had two episodes of seizure with altered mental status and was intubated for airway protection. The initial bicytopenia evolved to pancytopenia, with a white blood cell count of 3.9 × 10^3^/µL, hemoglobin 6.8 g/dL, and platelets 35 × 10^3^/µL. Her renal function continued to decline with a peak creatinine of 5.5 mg/dL, and she was started on hemodialysis. Her initial complement level (C3 and C4) counts were low, and she had multiple positive antibodies, including anti-double-stranded DNA, anti-Smith, anti-ribonucleoprotein, anti-Ro, and anti-La. The rheumatologic workup is shown in Tables 2, 3. A diagnosis of SLE was supported by the skin and hard palate ulcerations, pleuropericardial disease, and immunological workup. A presumptive diagnosis of HLH was supported by hyperferritinemia, hypertriglyceridemia, pancytopenia, lymphadenopathy, and severe hepatic inflammation. The initial HScore for reactive hemophagocytic syndrome showed a 70-80% probability of HLH with a total of 187 points.

**Table 2:** Rheumatological parameters in the hospital. SSA: anti-Ro; SSB: anti-La

Antibodies	Day 4	Day 33	Day 72	Reference range
SSA	>8.0	>8.0	-	0.0–0.9
SSB	2.8	0.6	-	0.0–0.9
Rho factor (IU/mL)	<0.10	-	-	0–15
Anti-Jo	<0.2	<0.2	-	0.0–0.9
Anti-DNA (IU/mL)	251	235	10	0–9
Anti-Smith	>8.0	>8.0	-	0.0–0.9
B2 microglobulin (mg/L)	17.2	-	-	0.6–2.4

**Table 3:** Complement parameters in the hospital.

Complement	Day 1	Day 3	Day 24	Day 32	Day 67	Day 70	Reference range
C3 (mg/dL)	<16	34	76	56	96	75	75–175
C4 (mg/dL)	-	<6	9	7	17	18	14–40

Rheumatology, Hematology/Oncology, Gastroenterology, Neurology, Cardiology, and Nephrology departments were consulted. She was started on high-dose pulse steroid therapy with immunosuppressive agents, including mycophenolate mofetil 250 mg daily and hydroxychloroquine 400 mg daily. She also received 14 sessions of plasmapheresis and 600 mg of rituximab twice with improvement in her clinical function. She was monitored for signs of infection while on an increased dose of steroids. During the course of her ICU stay, her mental status improved. Her renal function subsequently normalized and dialysis was stopped. The cytopenia resolved and the inflammatory indices including liver function tests and ferritin gradually returned to normal. The patient was weaned off the ventilator and a trach collar was placed. She was transferred to a long-term acute care facility for rehabilitation of muscle strength.

## Discussion

HLH is a rarely diagnosed, life-threatening condition that consists of a constellation of symptoms caused by cytokine storm and excessive immune activation [1]. The incidence of HLH has been reported to be one case per million persons per year [2]. HLH can occur as the familial primary form due to genetic defects leading to a decrease in the cytotoxic activity of natural killer (NK) cells or due to secondary etiologies, including infection, an autoimmune condition, malignancy, medications, and rheumatological conditions [1]. It usually affects children and young adults. HLH is characterized by immune dysregulation causing uncontrolled proliferation and activation of CD8 T-cells, lymphocytes, and macrophages, which causes hypersecretion of inflammatory cytokines [3]. The cardinal features include clinical and laboratory abnormalities, and consist of high-grade fever, hepatosplenomegaly, cytopenia (anemia, thrombocytopenia, and neutropenia), hyperferritinemia, hypertriglyceridemia, hypofibrinogenemia, reduced or absent NK cell activity, rapidly progressing multiorgan failure, and evidence of hemophagocytosis in the bone marrow [4].

HLH secondary to SLE is a rare clinical entity with an estimated prevalence of 0.9-4.6% [5]. The diagnosis of HLH secondary to SLE is complicated because they both have overlapping symptoms; however, HLH presents with features of hyperferritinemia, hypofibrinogenemia, and hypertriglyceridemia that are not present in SLE. Another aspect to consider is that HLH might lead to a delay in the diagnosis of SLE; hence, prompt immunological testing for SLE is advised in the setting of HLH to prevent diagnostic delay [2]. It has been reported that viruses may be implicated in the etiology of SLE. The phenomenon of molecular mimicry in which immune reactions against viral antigens may turn against the self-antigens leading to auto-immunity might be the cause of this association [6]. Limited data are available on the onset of clinical manifestations of SLE after infection with SARS-CoV-2; however, several studies have linked COVID-19 with other autoimmune diseases, including rheumatoid arthritis (RA) and multiple sclerosis. Joo et al. observed that viral infections including SARS-CoV-2 can increase the incidence of RA [7]. Tang et al. also reported a case of HLH after receiving a COVID-19 vaccine [8]. Autoantibodies, which are the hallmark of most autoimmune diseases, may also be detected in COVID-19 [9]. It has been speculated that SARS-CoV-2 can cause cross-reactivity with host cells which might trigger an autoimmune response [10]. SARS-CoV-2 increases tumor necrosis factor-alpha, interferon-gamma, interleukin (IL)-2, 6, 7, and 10, which show a form of secondary HLH [6]. Some of the clinical characteristics and laboratory findings observed in COVID-19 are reminiscent of HLH, including fever, lymphocytopenia, elevated LDH, D-dimer, ferritin, and rapidly progressing multiorgan failure. The cytokine profile observed in COVID-19 is also similar to HLH, with elevated levels of IL-2, 6, and 7 [4].

In our case, the patient presented with features of SLE after a few weeks of COVID-19 diagnosis. The patient was diagnosed with SLE based on the symptoms and immunological workup. As per the HLH-2008 diagnostic criteria, the diagnosis of HLH can be made by either fulfilling five out of eight diagnostic criteria or by molecular identification of HLH-associated gene mutation. In our case, the patient was diagnosed with HLH as she fulfilled five diagnostic criteria [11]. Most recently, a well-validated scoring system for the diagnosis of primary HLH was published, the HScore [12]. This scoring system incorporates cytopenia, fever, organomegaly, fibrinogen, ferritin, alanine aminotransferase, and hemophagocytosis, with an HScore of >250 consistent with 99% probability of HLH and <90 conferring <1% probability of HLH. The typical treatment for primary HLH includes etoposide, dexamethasone, cyclosporine, and the consideration of hematopoietic stem cell transplant [1]. The treatment of secondary HLH relies on the treatment of the primary disease. Literature has shown that rituximab may be an effective treatment option as an adjunct or monotherapy of HLH, especially in the setting of SLE by modulation of various parts of the cytokine cascade; however, further investigation needs to be done on the use of rituximab in HLH [13].

In the case of a diagnosis of HLH in a patient with COVID-19, it is empirical to control the hyperimmune response that leads to multiorgan failure and death. The effectiveness of the therapy is time-dependent to control the hyperactivated immune system. Corticosteroids, preferably dexamethasone, are the initial choice of treatment; however, plasma exchange can also be performed to eliminate cytokines and improve coagulation state [4]. If there is no improvement of symptoms in a patient with COVID-19, early goal-directed therapy might lead to improved outcomes [14]. A stepwise therapeutic approach with corticosteroids and immunosuppressive therapy might be required to reduce the effects of the cytokine storm and prevent the high mortality risk associated with HLH. Management by a multidisciplinary team, including a rheumatologist, intensivist, hematologist, and immunologist, might be needed to provide the patient with a full range of treatment options.

## Conclusions

Although single case reports have limitations, the present case shows a possible association of COVID-19 with the initial manifestation of SLE. Secondary HLH should be suspected in a patient who meets the diagnostic criteria of HLH. Early diagnosis and prompt intervention are required to reduce the mortality rate in patients in HLH. Further case reports are required to support this hypothesis.
